# Chinese botanical drugs for mitigating epidermal growth factor receptor-targeted therapy-associated cutaneous toxicity: immune mechanisms and translational pathways

**DOI:** 10.3389/fphar.2026.1931462

**Published:** 2026-09-22

**Authors:** Lu Lu, Hui Yu, Xinyang Yu, Wanlei Ren

**Affiliations:** Qingdao Central Hospital, University of Health and Rehabilitation Sciences, Qingdao, Shandong, China

**Keywords:** Chinese botanical drugs, cutaneous toxicity, EGFR-targeted therapy, oncodermatology, phenotype-stratified trials, skin barrier, translational medicine

## Abstract

**Background:**

Therapies targeting the epidermal growth factor receptor (EGFR) cause a spectrum of cutaneous toxicities that can compromise quality of life, treatment adherence, and cancer-treatment continuity. Persistent and phenotype-specific toxicities create a need for evidence-based adjunctive interventions.

**Purpose:**

Our aim was to appraise product-specific clinical and mechanistic evidence for Chinese botanical drugs in EGFR-targeted therapy-associated cutaneous toxicity and to define priorities for translational development.

**Methods:**

We conducted a structured narrative search of PubMed, Web of Science Core Collection, and Scopus from inception to 7 May 2026. Evidence was classified by the highest supporting design, from controlled human studies to indirect non-EGFR or computational evidence. We appraised directness, internal validity, product characterisation, outcome measurement, precision, and reporting completeness.

**Results and Discussion:**

Direct human evidence for defined Chinese botanical drugs remains insufficient to establish clinical efficacy. Available studies are limited by small samples, heterogeneous products, variable background care, and incomplete reporting. EGFR-specific experimental studies provide low-certainty support for barrier injury, inflammatory amplification, and phenotype-relevant mechanisms. Evidence from non-EGFR models, isolated metabolites, and network analysis remains hypothesis-generating.

**Conclusion:**

Defined Chinese botanical drugs should be evaluated only as adjuncts to standard supportive care. Progress will require standardised products, phenotype-stratified trials, prespecified patient-relevant and mechanistic outcomes, and complete reporting of safety and anticancer-treatment continuity.

## 1 Introduction

Epidermal growth factor receptor (EGFR)-targeted therapies improve outcomes in molecularly selected cancers, but their on-target cutaneous toxicities can impair quality of life, adherence, and treatment continuity. Clinically relevant phenotypes include papulopustular eruption, xerosis with fissuring, pruritus, periungual inflammation, hair changes, and persistent follicular inflammation. Their timing, morphology, and management differ across EGFR tyrosine kinase inhibitors (EGFR-TKIs), anti-EGFR antibodies, EGFR/mesenchymal-epithelial transition factor (MET) bispecific antibodies, and combination regimens (Basse et al., 2022; Vasiliki et al., 2026; Peng et al., 2018).

Cutaneous toxicity reflects disruption of EGFR-dependent skin homeostasis. EGFR blockade impairs keratinocyte differentiation and repair, weakens barrier and antimicrobial functions, and permits inflammatory amplification (Pastore et al., 2008; Pastore et al., 2014). For this review, these changes provide a practical framework for matching botanical candidates to phenotypes dominated by follicular inflammation, barrier failure, pruritus, or periungual disease (Figure 1).

**FIGURE 1:**
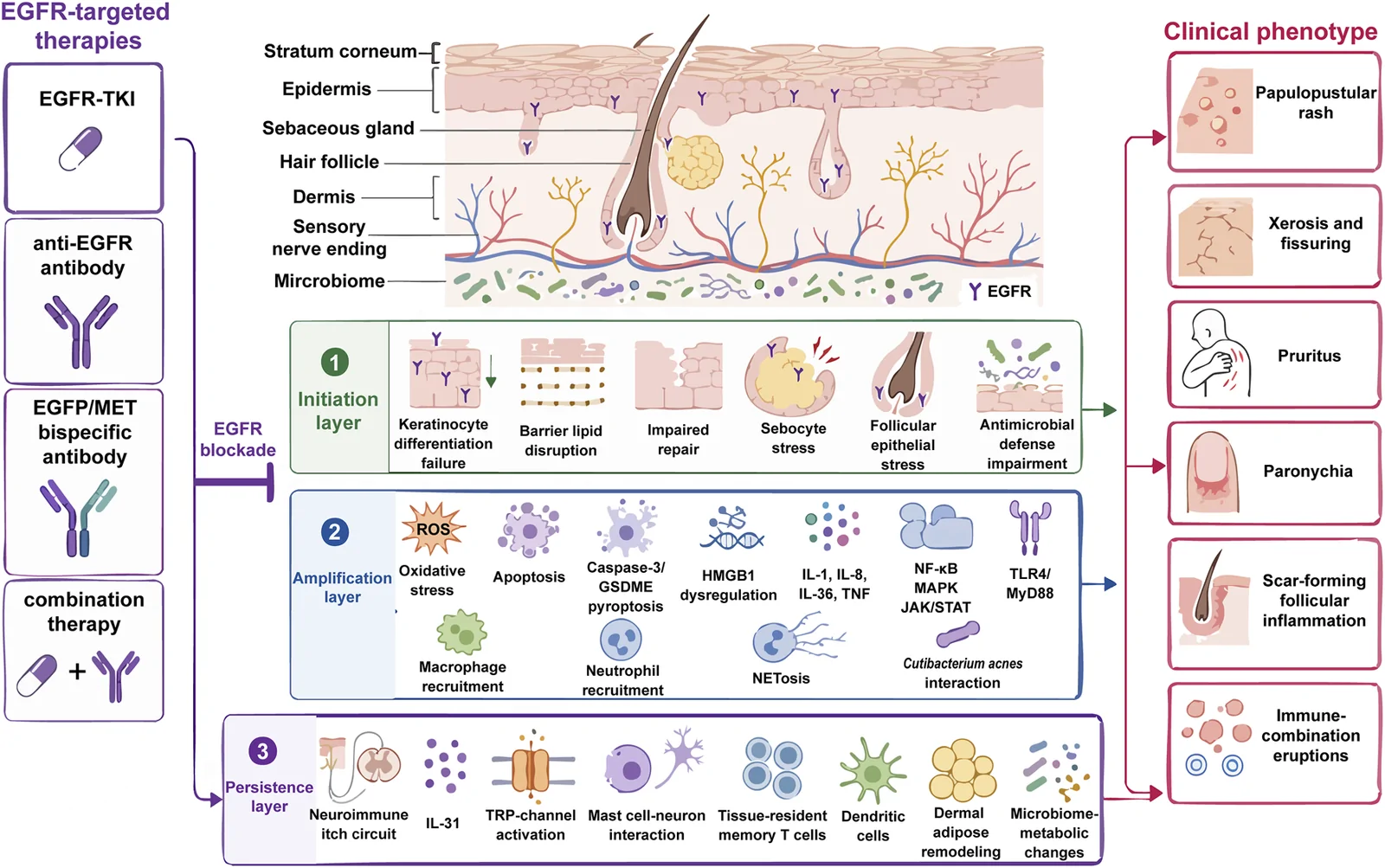
Layered epithelial-immune pathogenesis of epidermal growth factor receptor (EGFR)-targeted therapy-associated cutaneous toxicity.

Advances in EGFR-targeted drug design also broaden the range of molecular contexts in which cutaneous toxicity must be interpreted. Recent studies have described the design and synthesis of sulfonylated purine derivatives and sulfonyl hydrazone scaffolds as candidate EGFR inhibitors, using chemical characterisation, molecular docking, molecular-dynamics simulations, and drug-likeness or ADMET profiling (Saha et al., 2025; Rajaraman et al., 2025). A complementary study developed a radiolabelled peptide for multimodal imaging of the L858R-mutant EGFR, illustrating how mutation-aware molecular agents can support genotype-informed drug selection (Kim et al., 2024). These early-stage design and imaging studies do not establish clinical efficacy or cutaneous safety, but they are relevant to this review because inhibitor scaffolds, mutation selectivity, tissue distribution, and exposure may influence the phenotype and persistence of treatment-associated skin toxicity.

The broader cancer biology of EGFR signalling also shapes the clinical context in which EGFR-targeted toxicity is observed. EGFR/ErbB1 and ErbB2 signalling supports tumour-cell proliferation, invasion, angiogenesis, and metastasis, and peptide-based dual targeting of ErbB1 and ErbB2 has been explored as a strategy to interfere with these pathways (Patnaik et al., 2024). In *EGFR*-mutant non-small-cell lung cancer, immune features, mutation subtype, and the interaction between targeted therapy and immune-checkpoint treatment influence treatment selection and sequencing (Dong et al., 2024). Acquired resistance may involve histological transformation, including rare sarcomatoid conversion, with recurrent alterations in *TP53, RB1, MET*, and PI3K-related signalling (Pang et al., 2025). EGFR signalling also contributes to osteoclast activation and osteolytic bone metastasis, linking receptor biology to the tumour microenvironment beyond the primary lesion (Mohammad and Akhund, 2024). Recent work further suggests that CD24-directed immunotherapy may enhance responses to third-generation EGFR tyrosine kinase inhibitors in *EGFR*-mutated lung cancer (Liang et al., 2025). These cancer-focused findings do not directly establish mechanisms of cutaneous toxicity, but they underscore why drug generation, mutation context, resistance state, immune environment, and treatment combinations should be recorded when interpreting EGFR-associated dermatoses.

Although rash can correlate with antitumour response in some settings, it should not be viewed as a desirable treatment effect (Petrelli et al., 2012; Petrelli et al., 2013; Liu et al., 2013; Abdel-Rahman and Fouad, 2015). The clinical objective is to preserve anticancer dose intensity while limiting pain, itch, infection risk, and treatment interruption. Standard prophylaxis remains essential, but persistent xerosis, pruritus, recurrent follicular injury, paronychia, and combination-therapy eruptions support adjunctive, mechanism-informed interventions.

This review assesses Chinese botanical drugs only when the intervention is identifiable and directly relevant to EGFR-targeted therapy-associated cutaneous toxicity. It does not posit a system-level advantage for traditional Chinese medicine (TCM) over Ayurveda, European phytotherapy, or other traditional medical systems. Instead, each formula, topical preparation, or plant-derived metabolite is judged by product definition, direct clinical or experimental evidence, mechanistic plausibility, quality control, safety, and phenotype-specific outcomes.

## 2 Literature search and study selection

This review was informed by a structured narrative search of PubMed, Web of Science Core Collection, and Scopus from database inception to 7 May 2026. The search was designed to identify direct evidence for botanical interventions in EGFR-targeted therapy-associated cutaneous toxicity and to contextualise clinical phenotypes, mechanisms, and evidence gaps. Database-specific syntax was adapted from three concept blocks combined with AND: (EGFR OR “epidermal growth factor receptor” OR EGFR-TKI OR “EGFR tyrosine kinase inhibitor” OR cetuximab OR panitumumab OR amivantamab); (rash OR “skin toxicity” OR cutaneous OR dermatologic OR papulopustular OR xerosis OR pruritus OR paronychia); and (“traditional Chinese medicine” OR “Chinese herbal medicine” OR herbal OR botanical OR phytochemical OR honeysuckle OR glycyrrhizic acid). Reference lists of eligible reviews and primary studies were hand-searched for additional reports.

Eligible publications were English- or Chinese-language clinical studies, preclinical studies, translational studies, and high-quality reviews that addressed an EGFR-targeted therapy-associated cutaneous outcome. For intervention evidence, studies had to identify the botanical product, formulation, route, and intended use. Studies were excluded when they concerned non-EGFR anticancer toxicities, did not report a cutaneous outcome, examined an undefined mixture, were duplicate publications, or provided only general evidence from unrelated inflammatory dermatoses. Titles and abstracts were screened first, followed by full-text assessment against these criteria. Data were extracted on the intervention, design, population or model, comparator, outcome definition, follow-up, safety, and key limitations. Because the included literature was heterogeneous and largely exploratory, this was a structured narrative review rather than a systematic meta-analysis.

## 3 Cutaneous toxicities and therapeutic targets

### 3.1 Clinical phenotypes of EGFR-targeted therapy-associated skin toxicity

The term ‘acneiform’ is incomplete because EGFR-targeted therapy-associated toxicity includes more than a transient papulopustular eruption. Papules and pustules without comedones commonly affect seborrhoeic sites, whereas prolonged exposure may produce xerosis, fissuring, pruritus, periungual inflammation, and, less commonly, persistent follicular disease (Peng et al., 2018; Gorji et al., 2022; Nowaczyk et al., 2023). These distinctions are central to selecting relevant clinical endpoints for botanical interventions.

Phenotype assignment should account for the specific EGFR-directed agent, treatment timing, lesion distribution, evidence of infection, concurrent therapies, and possible immune-related adverse events. This assessment is necessary because a candidate intended to reduce neutrophil-rich papulopustular inflammation should not be evaluated as if it were a barrier-repair or antipruritic intervention (O'Reilly et al., 2024; Kale et al., 2025). Combination regimens warrant careful phenotype adjudication because immune checkpoint blockade broadens the differential diagnosis and compounds the clinical complexity of cutaneous toxicity (Iimura et al., 2026).

Rash may function as a pharmacodynamic marker in selected populations, but toxicity control should aim to maintain antitumour activity without making treatment dose limiting. Accordingly, studies of adjunctive botanical drugs should record anticancer treatment continuity and should not assume that reduced skin toxicity compromises efficacy (Petrelli et al., 2012; Abdel-Rahman and Fouad, 2015).

### 3.2 Barrier disruption and epithelial stress

EGFR blockade compromises keratinocyte proliferation, differentiation, migration, and repair, thereby producing a vulnerable epidermal barrier (Pastore et al., 2008; Franzke et al., 2012; Lichtenberger et al., 2013). This defect links xerosis and irritant sensitivity with inflammatory susceptibility and identifies barrier restoration as a relevant endpoint for botanical topical preparations.

Epithelial injury can be amplified by regulated cell death, oxidative stress, and cytokine release. EGFR-TKIs have been linked to caspase-3/gasdermin E-mediated pyroptosis in keratinocytes and sebocytes, while IL-8 and interleukin-1 (IL-1) signalling can promote inflammatory recruitment in experimental models (Zhu et al., 2025; Bangsgaard et al., 2012; Rodeck, 2009; Surguladze et al., 2009). These pathways are relevant only when a botanical candidate is tested against the corresponding phenotype and biomarker.

Follicular and microbial signals may further shape papulopustular toxicity. EGFR and mitogen-activated protein kinase kinase (MEK) inhibition can interact with *Cutibacterium acnes* to induce IL-36-associated inflammation (Satoh et al., 2020). Persistent itch or follicular injury may involve neuroimmune or JAK/STAT1-related pathways, but these mechanisms remain hypotheses for phenotype-specific intervention rather than indications for broad empirical treatment (Strobl et al., 2024; Li D. et al., 2025). Longitudinal human studies associate EGFR inhibition with skin dysbiosis and impaired barrier parameters. These data support microbiome-aware phenotyping but do not establish a causal role for any botanical intervention (Ashida et al., 2023; Saorin et al., 2022; Zhang et al., 2024).

EGFR-associated Janus kinase 2-signal transducer and activator of transcription 3 (JAK2-STAT3) signalling provides an additional mechanistic bridge, although current support comes mainly from tumour and non-cutaneous systems. A recent review describes EGFR-dependent STAT3 activation and identifies JAK2 as a facilitator of EGFR/EGFRvIII-driven STAT3 signalling and drug resistance (Lv et al., 2024). In EGFR-inhibitor dermatoses, this pathway should therefore be treated as a testable hypothesis rather than established skin-specific evidence. Confirmation requires drug-exposed keratinocyte, sebocyte, or follicular models and patient lesions, with phospho-JAK2 and phospho-STAT3 readouts linked to phenotype-specific outcomes.

## 4 Mechanistic basis for botanical interventions

### 4.1 Inflammation, microbiome, and metabolic persistence

Papulopustular toxicity is compatible with neutrophil-rich inflammation, including IL-8-dependent recruitment, and may therefore justify testing candidates with demonstrable effects on these pathways (Satoh et al., 2020; Bangsgaard et al., 2012). Evidence from other inflammatory dermatoses can generate such hypotheses, but it does not establish efficacy for EGFR-targeted therapy-associated rash.

Microbial composition, barrier status, and persistent immune activation may modify chronic or recurrent lesions. Their relevance in this setting is to guide endpoint selection, for example, lesion counts and inflammatory markers for papulopustular disease, or transepidermal water loss and itch scores for barrier- or pruritus-dominant disease.

### 4.2 Standard care and need for adjunctive therapies

Guidelines support proactive skin care, moisturizers, sunscreen, tetracycline-class antibiotics, topical anti-inflammatory treatment, and severity-guided treatment adjustment (Lacouture et al., 2010; Lacouture et al., 2011; Petrelli et al., 2016; Amitay-Laish et al., 2021). These measures are the foundation of care and the appropriate background treatment for trials of botanical drugs. In a phase 2 study, pre-emptive doxycycline supported protocolized prophylaxis and patient-reported outcome assessment in anti-EGFR-treated metastatic colorectal cancer. Such measures should form the background care for adjunctive-intervention trials (Ramirez-Daffos et al., 2022).

Important unmet needs remain in chronic pruritus, xerosis, recurrent follicular inflammation, periungual disease, and toxicity during combination therapy. Botanical interventions should therefore be developed as adjuncts to established supportive care and evaluated against phenotype-specific, patient-relevant outcomes rather than rash grade alone.

Mechanism-guided adjunctive approaches should be evaluated cautiously. For example, glycyrrhizic acid has been reported to attenuate osimertinib-associated cutaneous toxicity in preclinical work, but confirmatory clinical studies must establish the target phenotype, formulation, safety, and effect on anticancer treatment continuity.

## 5 Chinese botanical drugs: evidence and translational limitations

### 5.1 Direct clinical evidence

The available literature supports the feasibility of studying defined Chinese botanical drugs as adjunctive interventions for EGFR inhibitor-associated cutaneous toxicity. It does not establish clinical efficacy, a class effect for TCM, or superiority over standard supportive care. A 2019 meta-analysis reported possible benefit of Chinese botanical drugs for EGFR inhibitor-induced rash, but the underlying trials were generally small and heterogeneous (Chen et al., 2019). More recent reports include a single-centre prophylactic study (Li C. L. et al., 2022), a randomised study of honeysuckle therapy (Liu et al., 2022), an investigation of external Xiaozhen Zhiyang decoction (Lin et al., 2025), and a case report of Pien Tze Huang Unguentum Compositum (Zhang et al., 2022). Together, these studies provide a rationale for confirmatory trials of defined products, not for routine efficacy claims. The evidence hierarchy is therefore explicit: controlled human studies provide, at most, an efficacy signal; uncontrolled cohorts and case reports support feasibility or signal detection; and protocols provide no outcome evidence. A recent preclinical study of Xiao Zhen Fang identified PANoptosis-associated signals using untargeted metabolomics and transcriptomics. It supports evaluation of a chemically defined formula but does not provide evidence of clinical efficacy (Wang X. T. et al., 2026).

The certainty of the current clinical evidence is limited by small sample sizes, single-centre designs, heterogeneous formulations, inconsistent comparators, and variable background skin care. In several reports, key safeguards are insufficiently described, including allocation concealment, blinding of outcome assessment, adherence, rescue-medication rules, attrition, and prespecified analysis plans. These deficiencies create risks of selection, performance, detection, attrition, and selective-reporting bias. Subjective rash grading without blinded assessment, short follow-up, and incomplete recording of EGFR treatment interruption can further exaggerate apparent benefit or obscure harm. Differences in preparation, dose, route, and quality control also prevent valid pooling of effects across products.

Limitations also differ across individual reports. The double-blind afatinib pilot enrolled only 30 participants, its time-to-first-toxicity comparison was not statistically significant, and its later trial registration limits confidence in prospective outcome specification (Li C. L. et al., 2022). The three-arm honeysuckle study included multiple EGFR inhibitors and tumour types, while comparing prophylaxis with symptom-triggered treatment; this design confounds intervention effects with treatment timing, and overall rash incidence did not differ significantly (Liu et al., 2022). The Xiaozhen Zhiyang decoction cohort lacked a concurrent control group and assessed treatment over only 2 weeks, precluding separation of product effects from spontaneous improvement, co-interventions, or observer expectations (Lin et al., 2025). The Pien Tze Huang report describes one patient without a comparator, making efficacy, safety, and generalisability indeterminate (Zhang et al., 2022). Across these reports, incomplete botanical and batch-level characterisation further impedes attribution of outcomes to a reproducible material.

### 5.2 Ethnopharmacological context and material traceability

Ethnopharmacological context should guide candidate selection, not substitute for direct efficacy evidence. Honeysuckle (*Lonicera japonica* Thunb.) has been used in East Asian practice for inflammatory skin presentations. Xiaozhen Zhiyang decoction is a fixed external formula used for pruritic facial eruptions (Liu et al., 2022; Lin et al., 2025). Such uses may identify acceptable routes, symptoms, and feasible preparations. They do not establish activity against EGFR inhibitor-associated cutaneous toxicity.

To preserve this context during development, candidate products require two linked records. The botanical-material record (Pillar 2a) should report the accepted taxonomic name, authority, family, vernacular and pharmacopoeial names, plant part, origin, voucher, authentication, and processing. Pillar 2b should document formula composition and weights, source, batch, manufacturing standard, extraction, drug-to-extract ratio, marker metabolites, chemical fingerprint, contaminants, and stability. All formulation changes should trigger new material records and bridging analyses.

Where primary reports lack these data, the information should be recorded as unreported rather than inferred from a traditional name. Pien Tze Huang Unguentum Compositum contains non-botanical materials. It must therefore be considered a proprietary composite product, not evidence for a botanical class effect (Zhang et al., 2022). Future trials should link every clinical batch to its full material record, retain reference samples, and report quality-control failures alongside clinical outcomes.

### 5.3 Evidence appraisal and research priorities

Mechanistic and computational studies should be interpreted as hypothesis-generating. Network analysis can prioritise candidate plant-derived metabolites and pathways for a chemically characterised product, but it cannot validate efficacy or establish target engagement. Likewise, activity in atopic dermatitis, psoriasis, or generic inflammatory models cannot demonstrate benefit in EGFR inhibitor-associated toxicity. Evidence becomes mechanistically informative only when a defined product is tested in EGFR inhibitor-exposed keratinocyte, sebocyte, or follicular systems, or in drug-specific *in vivo* models, with dose-response data and phenotype-relevant readouts.

A major translational bottleneck is the scarcity of EGFR-inhibitor-specific preclinical models for botanical interventions. Most available studies use non-EGFR dermatosis models or unexposed keratinocytes, which cannot reproduce the drug, dose, exposure duration, sebaceous and follicular context, or treatment-continuity constraints of EGFR-targeted therapy. Priority models should include drug-exposed primary human keratinocyte-sebocyte-follicular co-cultures and clinically relevant *in vivo* systems, with authenticated products, exposure-matched dosing, vehicle and supportive-care controls, pharmacodynamic biomarkers, and phenotype-aligned readouts. Until such cell and animal models are established and independently validated, mechanistic claims should remain provisional and translation to patients should be regarded as a major unresolved bottleneck.

Evidence from non-EGFR inflammatory dermatoses can inform product-specific discovery but cannot serve as a surrogate for efficacy in EGFR-targeted therapy-associated toxicity. Studies of individual botanical drugs or plant-derived metabolites have reported effects on neutrophil extracellular traps, inflammatory signalling, or epidermal responses in atopic dermatitis and psoriasis models (Cheng et al., 2025; Hou et al., 2022; Hu et al., 2022; Huang et al., 2024; Huang et al., 2025). Comparable indirect signals have been reported for *Terminalia chebula* extract, Yu-Ping-Feng-San, Jiu-Wei-Yong-An Formula, modified Huang-Lian-Jie-Du decoction, Qingre Qushi formula, and Jingfang granules (Kim et al., 2022; Nie et al., 2024; Gu et al., 2022; Wang et al., 2022; Wang et al., 2024). These studies can prioritize preclinical testing but cannot establish benefit for an uncharacterized product or an EGFR-inhibitor rash.

Work in psoriasis and pustular disease identifies lineage-specific inflammation, NETosis, and keratinocyte signalling. It also shows why model transferability must be demonstrated rather than assumed (Feng et al., 2025; Li W. et al., 2025; McCluskey et al., 2022; Xu et al., 2023; Zhao et al., 2025). Material traceability must be coupled to chemical and biological verification. Databases, network-analysis frameworks, and formulation platforms can support candidate selection, but predictions require confirmation using authenticated materials, analytical fingerprints, and EGFR-relevant assays (Fang et al., 2021; Li et al., 2023; Zhao et al., 2023; Yang et al., 2024; Shen et al., 2024; Wang K. et al., 2025).

Broader research on barrier immunity, cutaneous metabolism, and tissue-resident immune memory informs the biological rationale for barrier-directed strategies. It supports phenotype-specific endpoints, rather than assuming that anti-inflammatory activity will correct all EGFR-inhibitor toxicities (Chambers and Vukmanovic-Stejic, 2020; Cibrian et al., 2020; Bangert et al., 2021; Poon et al., 2023; Zareie et al., 2025). Neuroimmune itch, sebaceous immunobiology, and microbial metabolites are plausible modifier pathways. Their measurement should remain exploratory unless linked to a prespecified phenotype and a validated assay (Dubin et al., 2021; Chiang et al., 2024; Xiao et al., 2023; Yang et al., 2020; Zouboulis et al., 2022).

Direct EGFR-injury studies are better suited to mechanism testing. Drug-specific models implicate sebocyte inflammatory activation, high mobility group box 1 (HMGB1) loss, epithelial oxidative or apoptotic injury, and epidermal repair responses, but none establishes clinical benefit for a botanical intervention (Ashida et al., 2016; Jiang et al., 2022; Liu et al., 2019; Mimura et al., 2024; You et al., 2024). Complementary rodent studies show that restoration of epidermal EGFR-associated signalling can attenuate experimental EGFR-inhibitor skin injury. This distinction is important because mechanism-matched preclinical efficacy does not demonstrate product-specific clinical benefit (Xie et al., 2022).

Other direct translational studies support restoring epidermal signalling or testing a defined intervention in EGFR-exposed systems. Their interpretation should remain conditional on intervention identity, model relevance, and human outcome data (Kim et al., 2020; Lacouture et al., 2021; Zheng et al., 2023; Wang C. et al., 2026; Choi et al., 2022; Li C. L. et al., 2022; Li Y. et al., 2022; Chen et al., 2022b; Wan et al., 2020; Zhou et al., 2025). Recent clinical and practice-oriented literature supports proactive multidisciplinary management that preserves anticancer treatment continuity. It does not justify replacing established supportive care with botanical drugs (Baas et al., 2012; Ceylan et al., 2026; Devireddy et al., 2025; Florez et al., 2025; Iimura and Kuroda, 2026). Direct clinical reports and protocols can refine eligibility criteria, prophylaxis timing, and outcome capture. Their heterogeneity also means that reports should specify the exact EGFR-directed regimen and concurrent supportive care (Hao et al., 2026; Hung et al., 2019; Kobayashi et al., 2015; Park et al., 2021; Yuan and Wang, 2023).

Patient experience, adherence, and real-world safety surveillance complement trial efficacy estimates. They help define chronic toxicity and treatment interruption but remain vulnerable to confounding and incomplete clinical detail (Jin et al., 2025; Nykaza et al., 2026; Skossyrskiy et al., 2025; Kafatos et al., 2020; Tseng et al., 2020). Bibliometric mapping of precision cancer medicine-related rashes can complement these priorities by identifying emerging research patterns (Zhao et al., 2022). Future protocols should incorporate microbiome sampling, supportive-care adherence, and oncodermatology expertise. Any botanical-drug efficacy claim should nevertheless meet a conservative evidentiary threshold (Ashida et al., 2023; Goncalo, 2023; Tran et al., 2025).

Reviews of *EGFR*-mutant tumour immunobiology and autoimmune-related skin disease offer cross-disciplinary context for candidate prioritization. Neither evidence stream can substitute for EGFR-specific efficacy studies (Dong et al., 2024; Wang Y. et al., 2025). Product reporting is itself a major source of uncertainty. Studies should specify botanical identity, plant part, extraction method, marker compounds, chemical fingerprint, batch consistency, contaminants, excipients, dose, route, stability, and storage. Without these details, a positive result cannot be reproduced, attributed to a defined exposure, or compared across studies. Oral products also require transparent assessment of hepatic and renal safety and potential interactions with EGFR-targeted agents, antibiotics, corticosteroids, and immunotherapies. Future trials should minimise bias through multicentre randomisation, an appropriate placebo or active-control strategy, standardised background prophylaxis, and blinded outcome assessment wherever feasible. Randomisation should be stratified by EGFR-directed agent, baseline skin phenotype, prior dermatological disease, and relevant combination therapy. Protocol registration and a prespecified statistical analysis plan are needed to reduce selective reporting. Analyses should report all randomised participants, missing-data handling, adherence, protocol deviations, rescue medication, and adverse events.

The primary endpoint should reflect the intended phenotype and should be assessed at a clinically meaningful time point. Papulopustular studies should report lesion counts and validated severity measures; barrier-dominant studies should include transepidermal water loss, fissure burden, and patient discomfort; pruritus studies should include itch intensity, sleep disturbance, and patient-reported outcomes. Rash grade alone is insufficient because it may not capture symptom burden, functional impairment, or durable benefit. Anticancer dose intensity, treatment interruption, and tumour outcomes should be collected to assess whether supportive benefit is achieved without compromising cancer treatment.

The immediate research priority is not broad screening of many formulas, but replication of the most plausible defined products in adequately powered, phenotype-stratified trials. Each candidate should demonstrate reproducible product quality, a prespecified biological rationale, a credible comparator, and a benefit beyond evidence-based supportive care. Negative and neutral studies should be reported with the same completeness as positive studies to reduce publication bias and permit balanced evidence synthesis.

### 5.4 Evidence hierarchy and quality assessment

To avoid conflating mechanistic plausibility with clinical efficacy, we assigned each claim to the highest level of supporting evidence. Tier 1 comprised controlled human studies in patients receiving EGFR-targeted therapy; Tier 2 comprised uncontrolled human studies, case series, and case reports; Tier 3 comprised EGFR-specific animal, *ex vivo*, or translational studies; Tier 4 comprised EGFR-specific *in vitro* studies; and Tier 5 comprised extract-level or isolated-metabolite experiments in non-EGFR models, network analysis, docking, or other computational predictions. Evidence was assessed across six prespecified domains: directness to EGFR-targeted cutaneous toxicity, internal validity, product characterisation, outcome validity, precision, and completeness of reporting. Overall confidence was rated as high, moderate, low, or very low and was downgraded for indirectness, bias, inconsistency, imprecision, or incomplete reporting.

The evidence matrix in Table 1 is a qualitative quality assessment rather than a pooled effect estimate. It distinguishes what the current literature can support from what remains unproven. Human studies can generate a clinical signal, but cannot establish routine efficacy when design safeguards, comparators, and product definitions are inadequate. EGFR-specific animal and cell studies can support a biological mechanism but cannot determine clinical benefit. Indirect botanical, extract-level, and computational findings can prioritise hypotheses only.

**TABLE 1:** Evidence hierarchy and qualitative quality assessment of proposed targets and mechanisms.

Proposed target or claim	Highest evidence tier in this review	Overall confidence	Critical interpretation
Clinical improvement of EGFR-targeted therapy-associated cutaneous toxicity by a defined Chinese botanical drug	Tier 1–2: limited randomised, single-centre, and uncontrolled human reports	Very low	Signals of feasibility only. Heterogeneous formulations, small samples, incomplete blinding and outcome reporting, and variable background care preclude routine efficacy claims
Barrier repair and epithelial injury	Tier 3–4: EGFR-specific preclinical and cellular evidence; limited human physiologic endpoints	Low for mechanism; very low for product efficacy	Supports a biologically plausible target for xerosis and barrier-dominant disease. It does not show that a botanical product restores barrier function or improves patient outcomes
Neutrophil-rich inflammation, IL-8, and IL-36	Tier 3–4: drug-specific experimental studies with limited clinical association	Low	Supports inflammatory plausibility in papulopustular disease. No defined botanical intervention has established target engagement or clinical efficacy through this axis
Pyroptosis and oxidative epithelial stress	Tier 3–4: EGFR-TKI cell and animal studies	Low	Identifies candidate mechanisms of epithelial injury. Translation is limited by scarce human tissue confirmation and absence of product-specific intervention trials
Microbiome, neuroimmune itch, and JAK/STAT-related persistence	Tier 4–5: mixed direct and largely indirect evidence	Very low	These pathways may modify phenotype or chronicity, but current botanical evidence is predominantly indirect and cannot support therapeutic attribution
Network analysis and multi-target predictions	Tier 5: computational or database-derived predictions	Very low	Useful for prioritisation only. Predictions require chemical verification, target-engagement evidence, and validation in EGFR-specific models before clinical inference

Network analysis should be positioned as a preliminary target-prediction and prioritisation tool rather than as mechanistic validation. Its outputs require triangulation through multi-omics analyses, cellular assays in drug-exposed keratinocyte, sebocyte, and follicular systems, and patient-derived biomarkers or lesion samples. Concordance across these layers, together with pharmacodynamic readouts and phenotype-specific clinical outcomes, is needed to support target engagement and translational inference.

Topical delivery platforms are enabling technologies, not evidence of clinical benefit. Hydrogels can provide local retention, hydration, and sustained release, whereas microemulsions can improve solubilisation, dose uniformity, and penetration of poorly water-soluble constituents. These advances may help control skin exposure and tolerability, but they require formulation-specific evaluation of release kinetics, permeation, irritation, stability, batch reproducibility, and performance in EGFR-relevant models before clinical inference. Their value should ultimately be tested against phenotype-specific outcomes and treatment continuity in patients receiving EGFR-targeted therapy.

## 6 Translational priorities for trial design

Future research should move from descriptive reports to phenotype-stratified, bias-resistant clinical studies. Standard prophylaxis and dermatology co-management should remain the background of care. Investigational botanical products should be evaluated as adjuncts, with the clinical question, target phenotype, comparator, and expected mechanism defined before enrolment. This approach separates established supportive measures from interventions that remain hypothesis generating. The phenotype-stratified translational framework in Figure 2 integrates candidate selection, mechanistic endpoints, and trial design requirements.

**FIGURE 2:**
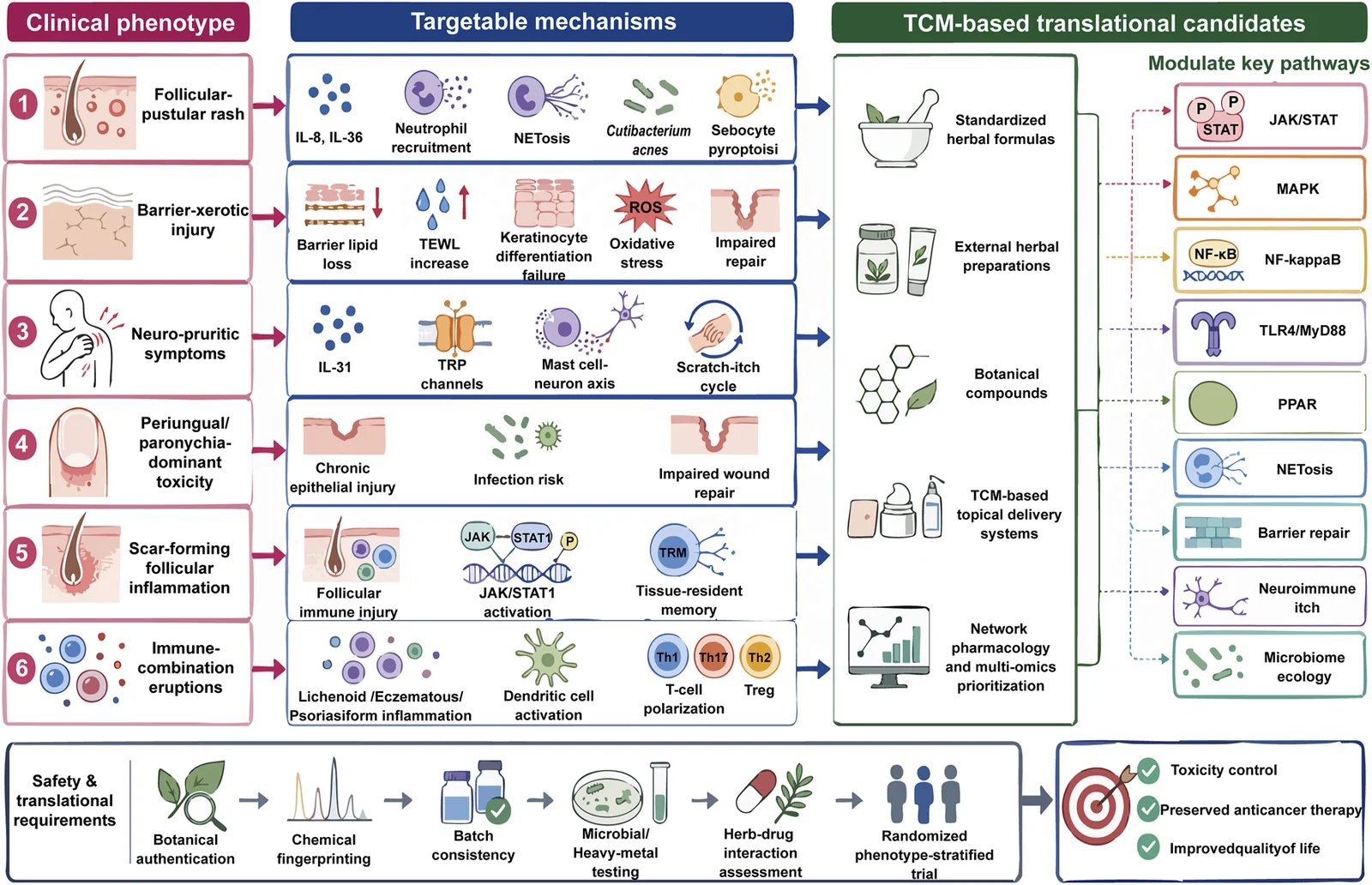
Phenotype-stratified translational and trial-design framework for Chinese botanical drug candidates in targeted therapy-associated cutaneous toxicity.

Risk and phenotype stratification should precede randomisation. Relevant variables include the EGFR-directed agent, dose and treatment duration, prior skin disease, baseline xerosis, concurrent therapies, and early toxicity. Endpoints should follow the dominant phenotype. Follicular-pustular studies should assess lesion counts, pustule scores, pain, infection, and antibiotic use. Barrier-xerotic studies should assess transepidermal water loss, corneometry, fissure scores, lipid markers, and discomfort. Pruritus-dominant studies should assess itch intensity, sleep disturbance, scratching behaviour, and neuroimmune markers. This design reduces outcome heterogeneity and improves interpretability.

Biomarker selection should follow the layered epithelial-immune model established in Section 2 and Figure 1, but the model is not restated here. For trial design, the relevant readouts are phenotype-linked: lesion counts, pustule scores, pain, infection and antibiotic use for follicular-pustular toxicity; transepidermal water loss, corneometry, fissure scores, lipid markers, and discomfort for barrier-xerotic disease; and itch intensity, sleep disturbance, scratching behaviour and neuroimmune markers for pruritus-dominant disease. Table 2 consolidates these endpoint and biomarker recommendations for botanical and other mechanism-guided interventions.

**TABLE 2:** Trial-design and biomarker checklist for phenotype-stratified intervention studies.

Trial domain	Recommended design element	Endpoint or biomarker package	Rationale and supporting citations
Eligibility and stratification	Stratify by drug class, baseline dermatologic history, early phenotype, combination immunotherapy exposure, and severity trajectory	Drug class, timing, Common Terminology Criteria for Adverse Events (CTCAE) grade, phenotype category, prior skin disease, concomitant antibiotics/corticosteroids, treatment continuity	Phenotype and drug-class heterogeneity are repeatedly emphasized in EGFR-TKI, antibody, amivantamab, and combination-therapy literature (Basse et al., 2022; Nykaza et al., 2026; Kale et al., 2025)
Intervention definition	Prespecify formula composition, route, dose, frequency, duration, rescue therapy, adherence method, and comparator arm	Botanical authentication, chemical fingerprinting, batch consistency, microbial/heavy-metal testing, stability, adverse-event capture	Standardization is essential because TCM studies vary in formulation, blinding, sample size, and endpoint quality (Chen et al., 2019; Li et al., 2022a; Zhao et al., 2023)
Objective skin measurement	Combine clinician grading with quantitative measures rather than relying only on global rash severity	Lesion counts, calibrated photographs, transepidermal water loss, corneometry, fissure score, dermoscopy, microbiome sampling when relevant	Barrier, follicular, microbial, and inflammatory mechanisms require phenotype-specific readouts (Lacouture et al., 2011; Satoh et al., 2020; Nicolaou and Kendall, 2024)
Patient-reported outcomes	Capture symptoms that drive adherence and quality of life, especially itch, pain, sleep disruption, and functional impairment	Itch numerical rating scale (NRS), sleep score, Dermatology Life Quality Index, pain/burning score, hand-foot or nail-function measures, rescue medication burden	Chronic itch and xerosis are clinically important and may reflect neuroimmune or barrier mechanisms not captured by rash grade alone (Dubin et al., 2021; Li et al., 2025a; Iimura et al., 2026)
Mechanistic biomarkers	Map candidate interventions to initiating, amplification, or persistence layers before efficacy claims are made	Keratinocyte apoptosis/pyroptosis markers, IL-1, IL-8, IL-36, JAK/STAT, mitogen-activated protein kinase (MAPK), nuclear factor kappa B (NF-kappaB), NETosis, lipidomics, TRP-channel or IL-31 markers	Mechanism-matched endpoints improve interpretability for pathway-directed topical agents and TCM candidates (Zhu et al., 2025; You et al., 2024; Wang C. et al., 2026)
Cancer-care safety	Assess whether dermatologic benefit preserves anticancer therapy without masking serious immune-mediated toxicity	Dose interruption, dose reduction, infection, systemic exposure, liver/renal events, oncology response context, irAE adjudication when applicable	The goal is toxicity control with treatment continuity, not suppression of useful pharmacodynamic signals or delayed recognition of immune toxicity (Petrelli et al., 2012; Abdel Rahman et al., 2015; Vasiliki et al., 2026)

## 7 Knowledge gaps and future directions

The main evidence gap is the lack of comparable, adequately reported studies. Research should prioritize risk prediction, phenotype definition, product standardisation, valid outcome measurement, and long-term safety. Trials should distinguish symptomatic relief from modification of epithelial injury and test whether an intervention adds benefit to evidence-based supportive care. Both positive and negative findings are needed to build a reliable evidence base.

Risk prediction remains underdeveloped. Early clinical observations, drug class, and dermatologic history are useful, but they do not explain why toxicity ranges from mild, transient eruptions to disabling chronic disease. Pharmacogenetic predictors have been explored for EGFR inhibitor-associated skin toxicity (Parmar et al., 2013). Human leukocyte antigen (HLA)-related susceptibility has also been reported in osimertinib hypersensitivity, which suggests that immune pathways may contribute to some reactions (Chen C. B. et al., 2025). Plasma biomarkers may help characterize inflammatory states before rash onset (Hichert et al., 2017). Future models should treat toxicity as drug- and phenotype-specific and integrate clinical, genetic, and molecular information. Their purpose should be to identify patients who need earlier prophylaxis, dermatology review, or closer surveillance, not to restrict access to effective cancer therapy.

Real-world evidence can complement clinical trials, which often exclude older adults, people with dermatologic comorbidities, and those receiving complex treatment sequences. A 20-year pharmacovigilance study provides long-term safety data for cetuximab and panitumumab across diverse populations (Jin et al., 2025). In non-small cell lung cancer, analysis of spontaneous reports in the US Food and Drug Administration (FDA) Adverse Event Reporting System can help distinguish class effects from drug-specific adverse-event patterns (Yu et al., 2026). Pharmacovigilance should record morphology, timing, concomitant medications, management, and outcomes, rather than adverse event counts alone. Treatment continuity is particularly important. In patients receiving osimertinib for *EGFR*-mutant non-small cell lung cancer, prophylactic skin-toxicity management may affect treatment continuity and clinical outcomes (Khongyot et al., 2026). Trials and registries should therefore report dose interruption, dose reduction, emergency visits, dermatology referrals, antibiotic and systemic steroid exposure, and patient-reported distress.

Biomarker development should be phenotype specific. Papulopustular rash may be assessed with IL-8, IL-36, neutrophil activation markers, microbial composition, and follicular inflammation scores (Satoh et al., 2020; Bangsgaard et al., 2012). Lipidomics, transepidermal water loss, corneocyte maturity, and barrier protein expression may be more informative for xerosis and barrier injury (Nicolaou and Kendall, 2024; Franzke et al., 2012). IL-31, mast-cell activity, and sensory-neuron mediators merit study in pruritus-dominant toxicity (Chen Y. et al., 2025; Li D. et al., 2025). JAK/STAT1 activation and tissue-resident memory signatures may be relevant to chronic follicular injury (Tokura et al., 2021; Strobl et al., 2024). A single marker is unlikely to be sufficient. A combined panel of phenotype, imaging, barrier measures, and inflammatory variables may be more informative.

Biomarker analyses should be exploratory unless they are prospectively specified and analytically validated. A change in a cytokine, microbiome feature, or barrier marker does not by itself demonstrate clinical benefit or mechanism. Biomarkers should be interpreted alongside phenotype-specific clinical outcomes, patient-reported symptoms, rescue treatment, and treatment continuity. This safeguards against overinterpretation of surrogate signals in small or uncontrolled studies.

Future work should connect early epithelial injury with long-term clinical outcomes. Epithelial cells can sense tissue damage and activate repair programs that are distinct from pathogen sensing (Liu et al., 2023). Repeated EGFR inhibition may therefore create persistent epithelial stress rather than a single acute wound. Because EGFR blockade impairs keratinocyte and follicular repair, inflammation may persist after the initial rash resolves. Studies should measure repair markers alongside inflammatory markers.

Barrier repair should be treated as a biological endpoint, not only as comfort care. Although trials commonly include moisturizers as background prophylaxis, they rarely measure barrier recovery. Protocols should include transepidermal water loss, lipid composition, corneocyte maturity, fissure scores, and patient-reported burning. These measures can help distinguish prevention of epithelial injury from symptomatic coverage and may explain severe xerosis without prominent pustules. Gefitinib-associated barrier impairment has been linked to ephrin type-A receptor 2 (EPHA2), which suggests that epithelial pathways beyond direct EGFR signalling may contribute to toxicity (Zhang et al., 2025). Lesional studies could examine EPHA2, a disintegrin and metalloproteinase 17 (ADAM17), JAK/STAT1, IL-36, and pyroptosis-related markers together to identify pathway-predominant subgroups.

Supportive-care studies must also address the relationship between rash control and antitumour activity. In head and neck cancer, rash correlated with survival during cetuximab-based therapy, which may reflect pharmacodynamic EGFR inhibition (Bonner et al., 2010). This association does not mean that rash should be maintained. The relevant clinical question is whether toxicity can be minimized without compromising antitumour activity. Supportive-care trials should therefore collect tumour response, dose intensity, and progression data where feasible.

A future randomized trial could compare standard prophylaxis plus placebo topical preparation with standard prophylaxis plus a standardized botanical topical preparation in high-risk EGFR-TKI recipients. Stratification should include drug type, prior skin disease, and baseline xerosis. A prespecified primary outcome could be the proportion of participants who develop grade 2 or higher rash by week 6, with blinded assessment where feasible. Secondary outcomes could include lesion counts, itch, Dermatology Life Quality Index, transepidermal water loss, rescue-medication use, EGFR-TKI interruption, adherence, and adverse events. Cytokines, microbiome, lipidomics, and digital imaging should remain exploratory unless the study is powered and analytically validated for these measures.

Long-term follow-up is needed because EGFR-targeted therapy often continues for months or years. Studies limited to 6 or 8 weeks may miss chronic xerosis, recurrent pruritus, periungual disease, pigmentary change, scarring follicular disease, cumulative antibiotic exposure, and delayed toxicity. Follow-up at 3, 6, and 13 months would better define durability, late harms, and the effect of skin-toxicity management on treatment continuity.

Consensus reporting standards for integrative dermatologic-oncology trials would improve comparability. Reports should describe the intervention and its quality control, baseline phenotype, cancer treatment, comparator, rescue-medication rules, outcome definitions, missing-data handling, adverse-event monitoring, and treatment continuity. They should also state whether the intervention is intended for prevention, early treatment, rescue therapy, chronic symptom control, or recurrence prevention. This information would make future studies auditable and suitable for evidence synthesis.

## 8 Conclusion

EGFR-targeted therapies can cause distinct cutaneous syndromes that impair quality of life and treatment continuity. The phenotype-oriented framework established in Section 2 and illustrated in Figure 1 should therefore be used to select clinical endpoints rather than reproduced in the conclusion. Standard prophylaxis remains the foundation of care, while chronic or phenotype-specific toxicity may require carefully tested adjunctive strategies. Tables 2, 3 provide the corresponding phenotype and trial-design guidance.

**TABLE 3:** Phenotype-oriented framework for epidermal growth factor receptor (EGFR)-targeted therapy-associated cutaneous toxicity.

Clinical phenotype	Dominant mechanistic emphasis	Suggested assessment endpoints	Representative supporting citations
Follicular-pustular rash	Follicular epithelial stress, sebaceous inflammation, interleukin-8 (IL-8)/interleukin-36 (IL-36) signalling, neutrophil recruitment, and *Cutibacterium acnes* interactions	Lesion count, pustule score, pain/burning, bacterial culture when indicated, IL-8 or neutrophil markers, antibiotic requirement	Bangsgaard et al., 2012; Satoh et al., 2020; Zouboulis et al., 2022; Jin et al., 2023; Zhu et al., 2025
Barrier-xerotic injury	Impaired keratinocyte differentiation, lipid-barrier disruption, reduced repair capacity, oxidative stress, and fissuring-prone epidermal fragility	Transepidermal water loss, corneometry, fissure score, xerosis grade, topical rescue use, patient-reported discomfort	Franzke et al., 2012; Lichtenberger et al., 2013; Nicolaou and Kendall, 2024; Morita et al., 2021
Neuro-pruritic symptoms	Barrier breakdown coupled to mast cell-neuron signalling, T helper 2 (Th2)-associated itch pathways, interleukin-31 (IL-31) biology, and transient receptor potential (TRP)-channel-related sensory activation	Numeric itch rating scale, sleep disturbance, scratch burden, quality-of-life indices, IL-31 or neuroimmune biomarkers where feasible	Dubin et al., 2021; Li et al., 2025a; Chen et al., 2025a
Periungual and paronychia-dominant toxicity	Chronic epithelial injury at mechanically stressed sites, secondary inflammation or infection risk, and impaired tissue repair	Paronychia grade, pain/function score, walking or hand-use limitation, culture when exudative, treatment interruption or dose modification	Lacouture et al., 2011; Peng et al., 2018; Jin et al., 2025; Vasiliki et al., 2026
Scar-forming follicular inflammation	Follicular immune injury, EGFR-deficiency-associated Janus kinase/signal transducer and activator of transcription 1 (JAK/STAT1) activation, and alopecia/folliculitis decalvans-like persistence	Scalp mapping, follicular inflammation score, alopecia progression, dermoscopy or photography, Janus kinase/signal transducer and activator of transcription (JAK/STAT)-related tissue markers	Nowaczyk et al., 2023; Strobl et al., 2024; Mimura et al., 2024
Immune-combination eruptions	Overlap between EGFR blockade and immune checkpoint inhibitor-related lichenoid, eczematous, psoriasiform, or immune-mediated inflammation	Morphology-directed diagnosis, biopsy when atypical or severe, immune-related adverse event (irAE) grading, steroid-sparing needs, oncology treatment continuity	O'Reilly et al., 2024; Kale et al., 2025; Ceylan et al., 2026

Defined Chinese botanical drug formulas, plant-derived metabolites, and topical preparations should be assessed with a product-specific, evidence-tiered framework. Current data support feasibility and hypothesis generation, but not routine efficacy claims, class-wide activity, or replacement of evidence-based skin care. Future trials require reproducible products, appropriate comparators, prespecified phenotype-specific endpoints, blinded assessment where feasible, and complete reporting of safety and treatment continuity. Reporting neutral and negative findings is equally important. This approach can distinguish a true adjunctive benefit from bias, regression to the mean, co-intervention effects, and selective reporting.
